# The suppression of HSPA8 attenuates NLRP3 ubiquitination through SKP2 to promote pyroptosis in sepsis-induced lung injury

**DOI:** 10.1186/s13578-024-01239-z

**Published:** 2024-05-02

**Authors:** Jinlian Liu, Ke Song, Bingqi Lin, Zhenfeng Chen, Yan Liu, Xianshuai Qiu, Qi He, Zirui Zuo, Xiaodan Yao, Xiaoxia Huang, Zhuanhua Liu, Zhifeng Liu, Qiaobing Huang, Xiaohua Guo

**Affiliations:** 1 Department of Pathophysiology, Guangdong Provincial Key Laboratory of Cardiac Function and Microcirculation, Guangdong Provincial Key Laboratory of Proteomics, State Key Laboratory of Organ Failure Research, School of Basic Medical Sciences, National Experimental Education Demonstration Center for Basic Medical Sciences, Southern Medical University, Guangzhou, 510515 China; 2 Department of Medicine intensive care unit , National Clinical Research Center for Geriatric Diseases (Chinese PLA General Hospital), General Hospital of Southern Theatre Command of PLA, Guangdong Branch Center, Guangzhou, Guangdong China; 3 School of Basic Medical Sciences, Southern Medical University, 1023 Shatai Road, Tonghe, Guangzhou, 510515 China

**Keywords:** HSPA8, SKP2, NLRP3 inflammasome, Pyroptosis, Alveolar epithelial cells, Acute lung injury

## Abstract

**Background:**

Acute lung injury (ALI) is strongly associated with hospitalization and mortality in patients with sepsis. Recent evidence suggests that pyroptosis mediated by NLRP3(NOD-, LRR- and pyrin domain-containing 3) inflammasome activation plays a key role in sepsis. However, the mechanism of NLRP3 inflammasome activation in sepsis-induced lung injury remains unclear.

**Results:**

in this study, we demonstrated that NLRP3 inflammasome was activated by the down-regulation of heat shock protein family A member 8 (HSPA8) in Lipopolysaccharide (LPS) and adenosine triphosphate (ATP)-treated mouse alveolar epithelial cells (AECs). Geranylgeranylacetone (GGA)-induced HSPA8 overexpression in cecum ligation and puncture (CLP) mice could significantly reduce systemic inflammatory response and mortality, effectively protect lung function, whilst HSPA8 inhibitor VER155008 aggravated this effect. The inhibition of HSPA8 was involved in sepsis induced acute lung injury by promoting pyroptosis of AECs. The down-regulation of HSPA8 activated NLRP3 inflammasome to mediate pyroptosis by promoting the degradation of E3 ubiquitin ligase S-phase kinase-associated protein 2 (SKP2). In addition, when stimulated by LPS and ATP, down-regulated SKP2 promoted pyroptosis of AECs by further attenuating ubiquitination of NLRP3. Adeno-associated virus 9-SKP2(AAV9-SKP2) could promote NLRP3 ubiquitination and degradation, alleviate lung injury and inhibit systemic inflammatory response in vivo.

**Conclusion:**

in summary, our study shows there is strong statistical evidence that the suppression of HSPA8 mediates alveolar epithelial pyroptosis by promoting the degradation of E3 ubiquitin ligase SKP2 and subsequently attenuating the ubiquitination of NLRP3 to activate the NLRP3 inflammasome, which provides a new perspective and therapeutic target for the treatment of sepsis-induced lung injury.

**Supplementary Information:**

The online version contains supplementary material available at 10.1186/s13578-024-01239-z.

## Introduction

Sepsis, defined as life-threatening multiple organ dysfunction resulting from dysregulated host response to infection, is the most common cause of death in intensive care units [1]. Sepsis leads to inflammation in almost every organ system in different degree, with ALI and acute respiratory distress syndrome (ARDS) progress the fastest and case fatality rate can be as high as 40% [2]. ALI is characterized by a persistent malignant inflammatory response caused by excessive host defense immune response and accumulation of a large number of inflammatory mediators in lung tissue. The main manifestations are acute inflammation, endothelial barrier dysfunction, and alveolar epithelial damage, leading to protein-rich pulmonary interstitial edema and infiltration of immune cells into the alveolar cavity [3]. At present, mechanical ventilation, anti-inflammatory drugs and fluid conservative therapy are the main treatment methods for acute lung injury. However, the effect is not satisfactory [4]. Therefore, inhibiting the inflammatory response and promoting repair of damaged cells would be a potential therapeutic strategy for acute lung injury caused by sepsis.

Heat shock homologous protein 71 kDa (Hsc70), also known as HSPA8, is a member of the heat shock protein 70 family (Hsp70). HSPA8 is a constitutively expressed chaperone protein with a variety of biological functions, including regulations of cell division, chaperone activity, signal transduction, transcription, translation control [5], especially in mediating the ubiquitination and degradation of various proteins. The lasted view is that HSPA8 also regulate ATP hydrolysis, anti-apoptosis, regulate cell cycle, and maintain cell stability [6–8]. Previous studies have shown that inhibition of HSPA8 attenuates spinal cord I/R injury by acting on the NF-κB/NLRP3 inflammasome pathway in astrocytes [5]. In addition, HSPA8 inducer GGA could induce PRMT1 ubiquitination and degradation through E3 ubiquitin ligase CHIP, thereby promoting the apoptosis of human osteosarcoma cells [9]. ATP is one of the essential molecules for the activation of NLRP3 inflammasome. It is not clear whether HSPA8 mediates lung injury by regulating NLRP3 inflammasome in sepsis.

The NLRP3 inflammasome is a multi-protein platform composed of NLRP3, ASC, and caspase-1, which plays an important role in host defense against pathogens. Upon stimulation, NLRP3 binds to ASC to form an inflammasome complex, leading to caspase-1 activation, which is subsequently proteolytic to activate the proinflammatory cytokines interleukin-1β (IL-1β) and IL-18, as well as the cytosolic protein GSDMD [10, 11]. It is increasingly clear that post­translational modifications are a key mechanism in regulating NLRP3 activation [12]. Among the numerous post-translational modifications, ubiquitination and deubiquitination play a crucial role in the degradation or activation of NLRP3 [13]. Additionally, Previous studies have shown that the E3 ubiquitin ligase Cbl-b binds to the K63-polyubiquitin chain by linking the ubiquitin-associated domain of the leucine-rich repeat domain of NLRP3, and targets K496 of NLRP3 for K48-linked ubiquitination and proteasome-mediated degradation [14]. As an innate proinflammatory pathway, NLRP3 inflammasome activation may lead to AECs damage and lung dysfunction. Therefore, we wondered whether HSPA8, as a major molecule in ubiquitin-protein degradation, has a regulatory role in NLRP3 ubiquitination modification during sepsis.

The SKP2- Skp1-Cullin-1-F-box (SCF) complex functions as an E3 ubiquitin ligase and consists of an invariant SKP1 element and a variable element SKP2 F-box protein. SKP2 contains an F-box domain that binds to SKP1. SKP2 is also rich in leucine-rich repeats that specifically recognize ubiquitinated substrates. Due to the variability of SKP2, its regulation will also directly affect E3 ubiquitinase activity. Therefore, SKP2 is the most important component of the SCF complex and also plays a major role in the regulation of substrate protein ubiquitination [15]. Studies have shown that SKP2 promotes the degradation of substrate protein P27 K48 by targeting its ubiquitination. Therefore, SKP2 plays an important role in suppressing tumor formation by inhibiting the cell cycle [16]. STRING website analysis showed that there was a protein-protein interaction between HSPA8 and the E3 ubiquitin ligase SKP2-SCF complex, and this interaction was mainly related to the positive regulation of protein ubiquitination and degradation. However, it still not clear whether SKP2 is involved in the process of NLRP3 ubiquitination and what is the underlying mechanism for the alteration of SKP2 activity in sepsis-induced lung injury.

This study aims at investigating the effect of HSPA8 signal activation in AECs on sepsis-induced lung injury, as well as exploring whether HSPA8 is involved in the regulation of E3 ubiquitin ligase SKP2 and NLRP3 inflammasome.

## Materials and methods

### Animals

C57BL/6 mice (weight 17–22 g, 6–8 weeks old) were purchased from the Animal Lab Center of Southern Medical University (Guangzhou, China). All animals were maintained in temperature-controlled conditions (temperature 23 ^◦^C–25 ^◦^C; humidity 50% ± 5%) with 12 h rhythm and fed with sterile food and water. All animal experiments were approved by the National Institutional Animal Care and Ethical Committee of Southern Medical University.

### CLP animal models

C57 mice were anesthetized with isoflurane inhalation and their limbs were fixed. An approximately 2 cm incision was made along the middle of the abdominal wall of the mouse. The cecum was found and gently pulled out to the mesenteric vessels. The proximal cecal stool was gently squeezed to fill the cecum. Mesenteric surface vessels were then isolated and ligated with a 5-gauge sterile silk thread at a distance of 1 cm from the distal cecum. The cecum wall at the midpoint between the ligation and the distal cecum was pierced with a 21G sterile needle, and a little stool was gently squeezed. The cecum was returned to the abdominal cavity and sutured layer by layer with sterile No.4 surgical suture, and the postoperative mice were resuscitated with normal saline 50 mL/kg. In the sham-operation group, the cecum was explored by laparotomy without ligation or puncture.

To evaluate the role of HSPA8 in septic lung injury, mice were randomly divided into Sham, CLP, CLP + natural saline (NS), and CLP + GGA groups, with 3 mice in each group. In the CLP + GGA group, 800 mg/Kg GGA was used to gavage mice 2 h in advance, CLP modeling was then performed. The samples were taken 24 h after modeling for Co- immunoprecipitation (Co-IP) and Western blot analysis.

To observe the effect of HSPA8 agonist and inhibitor on the survival rate of CLP mice, C57 mice were randomly divided into three groups: CLP + DMSO, CLP + GGA, and CLP + VER155008, with 9 mice in each group. The mice were treated with 800 mg/Kg GGA by gavage 2 h before treatment, and 20 mg/kg VER155008 was injected intraperitoneally. The control group were given 1%DMSO intraperitoneally. Finally, a CLP model was established in each group of mice, the time of death in each group was recorded, and the survival rate was calculated.

### Construction and transfection of adeno-associated virus

AAV9-HSPA8 (NM_031165) and AAV9-SKP2 (NM_013787) overexpressing adeno-associated viruses were constructed and tested by Genechem (Shangha, China). Normal C57 mice were pretreated with 1*10^11 v.g for 14 days, and then the CLP model was established according to the group. Lung tissue, alveolar lavage fluid and peripheral serum were collected 24 h after operation for subsequent detection. The expression efficiency was detected by Western blot and immunofluorescence (Fig.S4C-D; Fig.S5B-C).

### Cell culture

Murine Lung Epithelial-12(MLE12, purchased from ATCC) cells. MLE12 cells were cultured in Dulbecco Modified Eagle medium (DMEM, Gibco, Grand Island, USA) supplemented with 10% fetal bovine serum and 1% streptomycin/penicillin in a 37 ° C incubator containing 5% CO2. MLE12 cells were stimulated with LPS (10 µg/mL) for 24 h, and 5mM ATP was added to the new medium for 1 h to establish the cell model.

### Isolation of primary ACE II

Mice were sacrificed by cervical dislocation and immersed in 75% alcohol for 5 min. The abdominal cavity was opened under sterile conditions, the bronchi were intubated, and the lungs were whitened by perfusion of PBS through the right ventricle to remove blood from the lungs. The lung tissue was then removed as soon as possible, trachea, bronchi, blood vessels and other tissues were removed in precooled PBS containing 1% streptomycin/penicillin. The PBS was washed repeatedly until clear.

The lung tissue was cut into 1 mm*1 mm*1 mm pieces and transferred to a 15mL centrifuge tube with 1 g/L trypsin added. The tube was incubated at 37 °C for 15 min, the digested cell suspension was transferred out, and an equal volume of DMEM medium containing 1% streptomycin/penicillin and 10% FBS was added to abrogate digestion.

Digestion of the remaining lung tissue was continued with trypsin and then incubated at 37 ° C for 15 min to remove the digestive fluid and stop digestion. Type I collagenase 1 g/L (2mL/ mouse) was added to the remaining lung tissue and incubated at 37 ° C for 15 min. The digestion solution was removed and the digestion was stopped with DMEM medium. The cell suspensions collected above were pooled and filtered through a 70 μm filter, followed by centrifugation at 1200 rpm/min for 5 min. Cells were transferred to IgG-coated dishes and incubated at 37 ° C for 60 min.

The suspension was aspirated and inoculated into another IgG-coated petri dish. The above step was repeated, and the non-adherent cells were aspirated, centrifuged at 1200 rpm/min for 5 min, seeded with cells, and then changed 2 to 3 times for further testing.

### Plasmid transfection

The plasmid of overexpression HSPA8 and Myc-SKP2 and its negative control plasmid vector (pcDNA3.1) were constructed by GenePharma (Shanghai, China). The constructed plasmids were confirmed by amplification and sequencing. MLE12 and primary ACE II were then transfected with Lipo3000 reagent according to the manufacturer’s instructions. After 8 h, cells were changed to fresh medium containing 10% FBS for 48 h and then stimulated with LPS for 24 h. The expression efficiency was detected by Western blot (Fig.S4A-B).

### RNA interference assay

An active oligonucleotide siRNA against HSPA8 or SKP2 was used for knockdown of HSPA8 or SKP2, with a scramble siRNA used as a Negative control. siRNA was transfected into MLE12 cells or primary ACE II with siRNA-Mate transfection reagent as indicated for 48 h and stimulated with LPS for 24 h, and the expression efficiency was determined by Western blot (Fig.S5A). Supplement Table 1 shows sequences of siRNAs against HSPA8 and SKP2.

### Western blot

Proteins (30 µg) were extracted from lung tissue, MLE12 cells, and primary ACE II and separated by electrophoresis on a 10–12% SDS-polyacrylamide gel. The separated proteins were then transferred to a PVDF membrane. Membranes were blocked with TBST containing 5% skim milk powder for 1 h at room temperature. The membranes were incubated with the corresponding primary antibody overnight at 4 ° C and with horseradish peroxidase-coupled (HRP) secondary antibody for 1 h at room temperature. Specific bands were detected using Pierce ECL western blotting substrate. All samples were normalized by GAPDH and analyzed using ImageJ software.

### Co-IP analysis

For Co-IP, whole cell and lung tissue extracts were lysed in IP buffer containing 1% phosphatase inhibitors, protease inhibitors, and PMSF. After centrifugation at 12,000 g for 15 min, the supernatant was removed and incubated overnight at 4 ° C with the corresponding IP monoclonal antibody, followed by Protein A + G magnetic beads for 3 h. Magnetic beads bound to proteins and antibodies were washed three to five times repeatedly with IP lysates. IP was denatured by boiling elution of IP lysate containing 1%SDS loading buffer. The samples collected were stored in the − 80 °C or -20 °C refrigerator for subsequent Western blot analysis.

### Quantitative real-time PCR (qRT-PCR)

Total RNA was extracted from MLE12 cells and lung tissue by Trizol reagent. 500ng RNA was reverse transcribed into cDNA using the HiScript III 1st Strand cDNA Synthesis Kit (+ gDNA wiper) kit, where genomic DNA was removed by DNase. Real-time qPCR was performed using SYBR Green Master Real-time PCR on a 7500 Real-Time PCR System (Applied Biosystems). The Ct value of β-actin was used as an internal reference for NLRP3, IL-1β, Caspase1, Caspase11, HMGB1, IL-1α, TNF-α、IL-6 and IL-1β mRNA in each sample. The qPCR primer sequences are shown in supplement Table 2.

### Histopathology

Fresh lung tissues were fixed with 4% paraformaldehyde, embedded in paraffin after 24 h, and 4 μm sections were taken for hematoxylin and eosin (H&E) staining. The sections were placed under a light microscope for imaging(Carl Zeiss, Germany). Lung injury score was performed with reference to previous literature [18]: 0, no injury; 1, 25% injury; 2, 50% injury; 3, 75% injury; and 4, 100% injury. Each injury was scored in ten randomly selected fields (200×) from each slide.

### Immunohistochemistry (IHC)

For immunohistochemical staining of HSPA8 in lung tissue, lung tissue sections were deparaffinized and antigen repaired, and nonspecific binding sites and endogenous peroxidase were blocked with 5% BSA and 0.3% H_2_O_2_, respectively. Lung tissue sections were incubated with HSPA8 primary antibody (1:200) overnight at 4 ° C, with goat anti-rabbit IgG for 1 h at room temperature, incubated with DAB (Dako, K5007), counterstained with hematoxylin, and dehydrated in ethanol. Finally, the sections were observed under a light microscope (Carl Zeiss Germany).

### Immunofluorescence staining

MLE12 cells and primary ACE II in confocal dishes were fixed with 4% paraformaldehyde for 15 min at room temperature, permeabilized with 0.1% Tritonx-100 for 15 min, and blocked with goat serum for 1 h at room temperature. Next, cells were incubated with respective primary antibodies overnight at 4 °C and stained with Alexa Flour 488 or Alexa Flour 555 conjugated secondary antibodies for 1 h at room temperature in the dark, followed by DAPI (1:100) staining for 10 min. The cells were washed three times with PBS buffer for 10 min between each step. Finally, the stained cells were observed under a laser microscope.

### Elisa

Mouse alveolar lavage fluid and serum were collected, and mouse IL-1β, TNF-α and IL-6 were detected by ELISA kit (R&D, USA) according to the instructions.

### Statistical analysis

All data were analyzed by GraphPad Prism 8.0 software and presented as the mean ± SEM. The differences between groups were evaluated by two-tailed unpaired Student’s t-test and one way analysis of variance with the least significant difference (LSD) test or Dunnett T3 test for post hoc comparisons. P-values less than 0.05 were considered statistically significant.

## Results

### Down-regulation of HSPA8 promotes AECs damage in sepsis

**Fig. 1:**
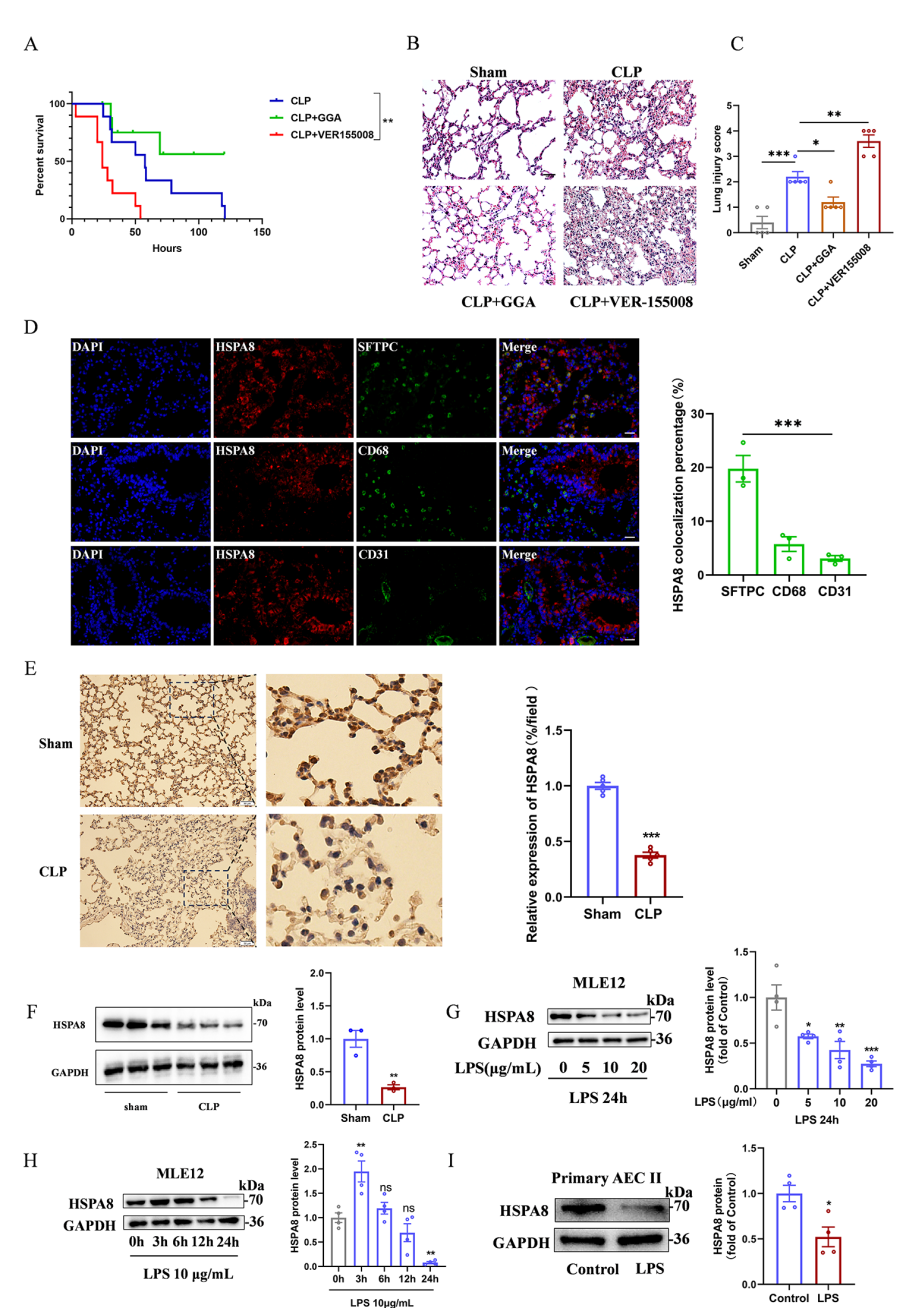
The down-regulation of HSPA8 mediates the injury of AECs in sepsis. (**A**) Survival analysis of CLP mice treated with HSPA8 agonist GGA (800 mg/Kg, ig) and inhibitor VER55008 (20 mg/Kg, ip) (*n* = 9). (**B**) Representative H&E staining and histological scores of lung section in Sham, CLP, GGA, VER155008 treated mice (*n* = 5, scale = 100 μm). (**C**) Histological scores of lung section in Sham, CLP, GGA, VER155008 treated mice (*n* = 5). (**D**) Representative double-staining immunofluorescence of AEC II (SFTPC), macrophages (CD68), endothelial cells (CD31) with HSPA8 in CLP group (*n* = 3, scale = 50 μm). (**E**) Representative images of immunohistochemical (IHC) for HSPA8 protein in Sham and CLP (*n* = 5, scale = 50 μm). (**F**) Western blot analysis of HSPA8 protein expression in lung tissue of sham and CLP groups (*n* = 3). (**G**) Western blot analysis of HSPA8 protein expression in MLE12 cells treated with different concentrations of LPS (0, 5, 10, 20 µg/ml) for 24 h (*n* = 4). (**H**) Western blot analysis of HSPA8 protein expression in MLE12 cells treated with 10ug/ml LPS after 0 h, 3 h, 6 h, 12 h and 24 h, respectively (*n* = 4). (**I**) Western blot analysis of HSPA8 protein expression in primary AEC II in Control and LPS-stimulated groups (*n* = 4). Data are expressed as mean ± SEM, *p < 0.05, **p < 0.01, ***p < 0.001, ns means no significance

HSPA8 agonist GGA and inhibitor VER155008 were used to investigate the role of HSPA8 in sepsis-induced lung injury. GGA improved the survival rate of CLP mice, while VER155008 accelerated the death of CLP mice(Fig. 1A). Pathological sections of lung tissue in the VER155008 group showed multiple alveolar cavities of different sizes, marked interstitial hyperemia and edema, and a large number of inflammatory cells and red blood cells infiltrated (Fig. 1B), as confirmed by lung injury scores (Fig. 1C). Immunofluorescence suggested that HSPA8 was widely expressed in primary type II alveolar epithelial cells (AEC II) compared with macrophages and endothelial cells in CLP mice (Fig. 1D). The data indicates that HSPA8 may mediate the occurrence of sepsis by damaging alveolar epithelial cells.

Next, we examined the expression levels of HSPA8 in septic lung injury. At animal level, immunohistochemical of lung sections showed that HSPA8 protein levels in lung tissues of mice at 24 h after CLP were significantly decreased (Fig. 1E). Consistent results were obtained by Western blot analysis of lung tissue (Fig. 1F). Meanwhile, to detect the protein level of HSPA8 in alveolar epithelial cells during sepsis, lung tissues from Sham and CLP mice were obtained for immunofluorescence. The results showed that level of HSPA8 in alveolar epithelial cells in lung tissue of CLP mice was significantly decreased compared with Sham (Fig.S1A). At the level of MLE12 cells, the expression level of HSPA8 decreased most significantly when MLE12 cells were stimulated with 20 µg/mL LPS for 24 h (Fig. 1G-H). The HSPA8 protein level in primary AEC II extracted from lung tissue of C57 mice (Fig. S1B) was detected by LPS stimulation, which was consistent with that in MLE12 cells (Fig. 1I). The results showe that HSPA8 protein levels are down-regulated in sepsis.

### In sepsis, the inhibition of HSPA8 mediates the occurrence of pyroptosis by affecting the level of GSDMD-N

**Fig. 2:**
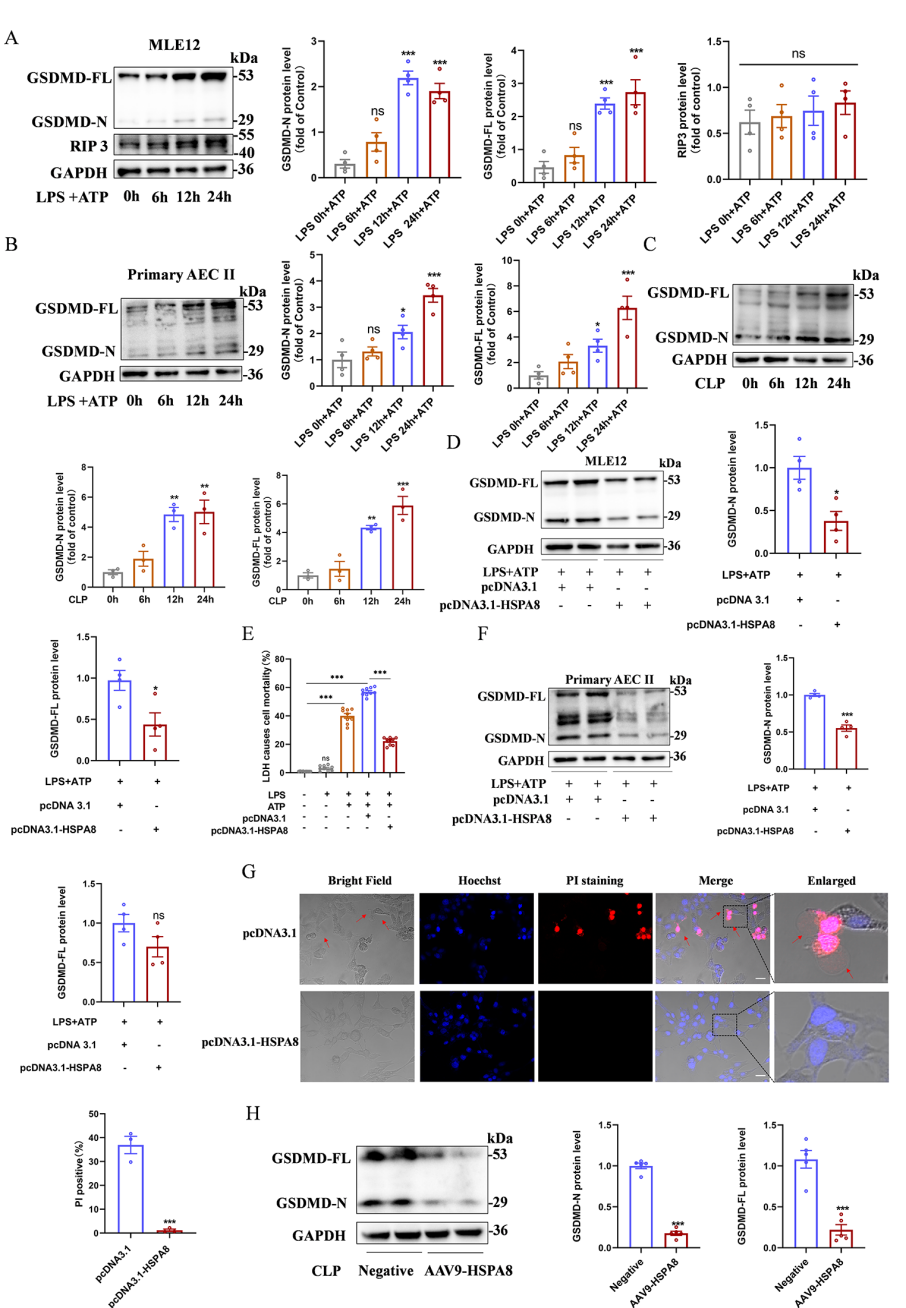
The down-regulation of HSPA8 mediates pyroptosis by affecting the level of GSDMD-N. (**A**) Western blot analysis of GSDMD and RIP3 protein expression in MLE12 cells stimulated with LPS + ATP at different time points (0 h, 6 h, 12 h, and 24 h) (*n* = 4). (**B**) Western blot analysis of GSDMD protein expression in primary AEC II stimulated by LPS + ATP at different time points (*n* = 4). (**C**) Western blot analysis of GSDMD protein expression at 0 h, 6 h, 12 h, and 24 h after CLP (*n* = 3). (**D**) Western blot analysis of GSDMD-N protein expression in MLE12 cells after overexpression of HSPA8 plasmid (*n* = 4), and lane1 and lane2 are independent repeats. (**E**) Comparison of LDH release levels in MLE12 cells treated with control, LPS, LPS + ATP, LPS + ATP + pcDNA3.1 and LPS + ATP + pcDNA3.1-HSPA8 (*n* = 9). (**F**) Western blot analysis of GSDMD-N protein expression in primary ACE II after overexpression of HSPA8 plasmid (*n* = 4), and lane1 and lane2 are independent repeats. (**G**) Representative confocal images and percentage of PI positive MLE12 cells in pcDNA3.1 and pcDNA3.1-HSPA8 (*n* = 3, scale = 10 μm). (**H**) Western blot analysis of GSDMD-N protein expression in CLP mice infected with AAV9-HSPA8 (*n* = 5). Data are expressed as mean ± SEM, *p < 0.05, **p < 0.01, ***p < 0.001, ns means no significance

Hoechst33342/PI staining showed that there was no significant difference in cell apoptosis among Ctrl, LPS, ATP and LPS + ATP, while the LPS + ATP group showed obvious cell death (Fig. S2A). Cell death includes pyroptosis, ferroptosis and inflammatory necrosis, and pyroptosis is the most significant type of cell death stimulated by LPS + ATP. To assess the type of cell death in MLE12 cells, the protein level of gasdermin D (GSDMD) and receptor-interacting protein 3 (RIP3) in MLE12 cells stimulated with LPS + ATP at 0 h, 6 h, 12 h, and 24 h were measured by Western blot. The results showed that the expression level of GSDMD-N and GSDMD-FL were significantly increased after LPS + ATP stimulation for 12 h and 24 h, while RIP3 was not different among the groups (Fig. 2A). The same results were obtained in primary AEC II and at the animal level. (Fig. 2B-C). LPS + ATP significantly increased the mRNA levels of TNF-α, IL-6 and IL-1β in MLE12 cells (Fig.S2B). Furthermore, we also examined the release levels of the three inflammatory factors in broncho alveolar lavage fluid (BALF) and serum of CLP mice and obtained consistent results (Fig.S2C-D). This suggests that alveolar epithelial cells mediate lung injury through pyroptosis in sepsis.

MLE12 cells and primary AEC II were transfected with HSPA8 overexpression plasmid to study the relationship between HSPA8 and pyroptosis. Western blot suggested that compared with the control group, GSDMD-N and GSDMD-FL level in the pcDNA3.1-HSPA8 group were significantly decreased (Fig. 2D and F). The release level of LDH in LPS + ATP + pcDNA3.1-HSPA8 group was significantly lower than that in pcDNA3.1 and LPS + ATP group (Fig. 2E). Confocal laser scanning microscope showed that the positive rate of pyroptosis vesicles and PI in pcDNA3.1-HSPA8 group was significantly reduced (Fig. 2G). Furthermore, we demonstrated that AAV9-HSPA8 could significantly reduce the protein levels of GSDMD-N and GSDMD-FL in the lung tissues of CLP mice (Fig. 2H). In conclusion, overexpression of HSPA8 inhibited the occurrence of pyroptosis by affecting the protein level of GSDMD-N.

In sepsis, down-regulation of HSPA8 mediates the occurrence of AECs pyroptosis by activating the NLRP3 inflammasome.

**Fig. 3:**
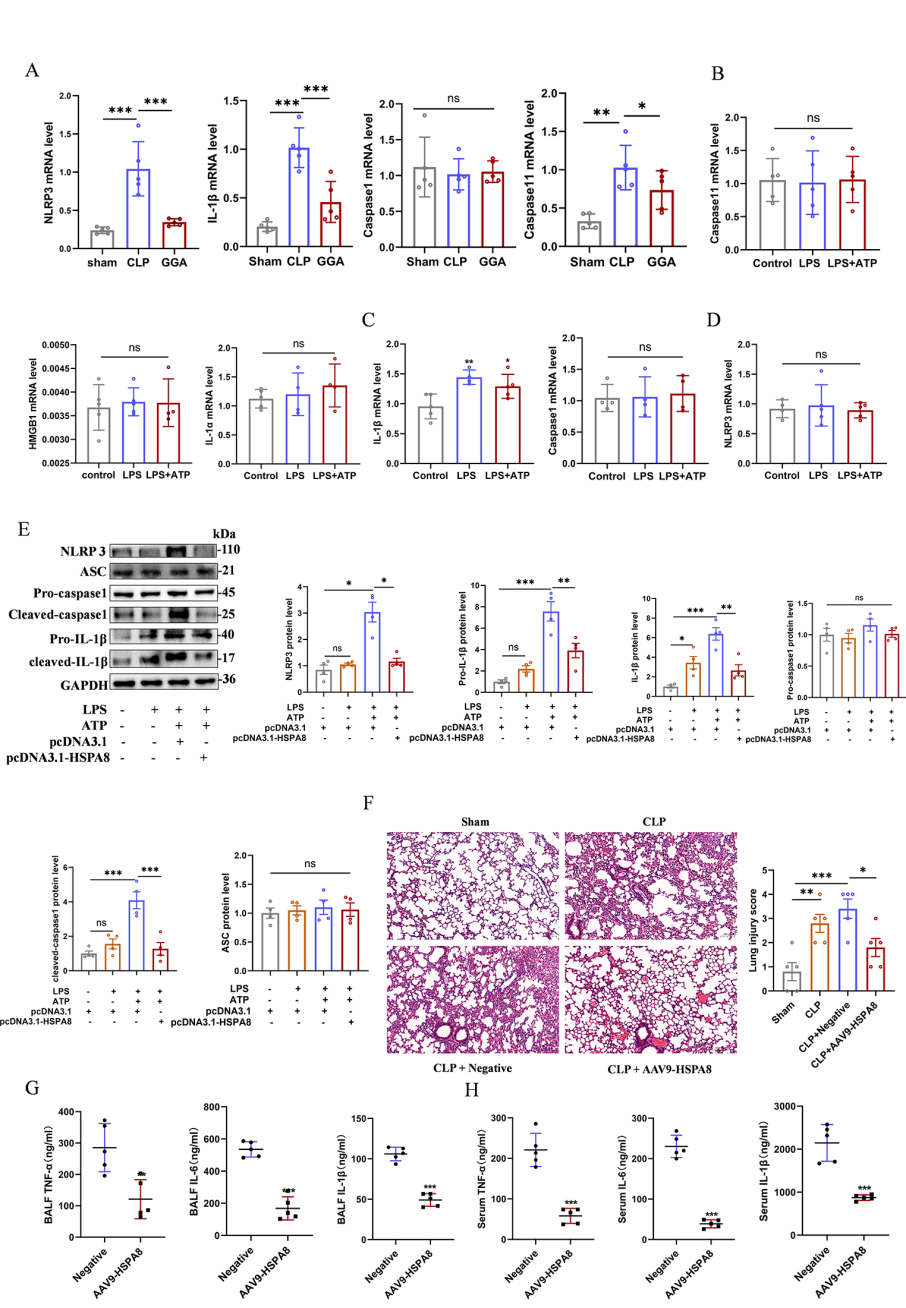
In sepsis, the suppression of HSPA8 mediates the occurrence of classical pyroptosis by activating the NLRP3 inflammasome. (**A**) mRNA levels of NLRP3, IL-1β, Caspase1 and Caspase11 in lung tissue of Sham, CLP and GGA induced mice (*n* = 5). (**B**) mRNA levels of Caspase11, HMGB1 and IL-1α in MLE12 cells stimulated by LPS and LPS + ATP (*n* = 5). (**C**) mRNA levels of IL-1β and Caspase1 in MLE12 cells stimulated by LPS and LPS + ATP (*n* = 5). (**D**) mRNA levels of NLRP3 in MLE12 cells stimulated by LPS and LPS + ATP (*n* = 5). (**E**) Western blot analysis of NLRP3 inflammasome related protein expression in MLE12 cells after overexpression of HSPA8 plasmid (*n* = 5). (**F**) Representative H&E staining and histological scores of lung section in Sham, CLP, negative, and AAV9-HSPA8 (*n* = 5, scale = 100 μm). (**G**) ELISA analysis of BALF TNF-α, IL-6 and IL-1β levels in negative and AAV9 - HSPA8 group (*n* = 5). (**H**) ELISA analysis of serum TNF-α, IL-6 and IL-1β levels in negative and AAV9 - HSPA8 group (*n* = 5). Data are expressed as mean ± SEM, *p < 0.05, **p < 0.01, ***p < 0.001, ns means no significance

To investigate the specific mode of lung pyroptosis in septic lung injury, RNA was extracted from lung tissue of CLP mice treated with HSPA8 agonist GGA for qRT-PCR analysis. The results showed that compared with Sham group, the mRNA level of NLRP3, IL-1β, and Caspase11 in CLP group were significantly increased, and GGA could significantly reduce the mRNA level of these indicators. There was no significant difference in Caspase1 mRNA level among the three groups (Fig. 3A). There was no significant difference in the mRNA levels of Caspase11, HMGB1, IL-1α, Caspase1 and NLRP3 in MLE12 cells stimulated by LPS + ATP, while the level of IL-1β was significantly increased (Fig. 3B-D). Western blot suggested that the protein expression of NLRP3, Cleaved-Caspase1, Pro-IL-1β and Cleaved-IL-1β in the LPS + ATP group were significantly higher than those in the LPS group. However, pcDNA3.1-HSPA8 group could significantly reduce these proteins level, and there were no significant differences in ASC and Pro-Caspase1 among all groups (Fig. 3E). AAV9-HSPA8 significantly alleviated lung injury in CLP mice (Fig. 3F) and reduced release of TNF-α, IL-6, and IL-1β in BALF and serum (Fig. 3G-H). These data suggest that downregulation of HSPA8 mediates classical pyroptosis in sepsis and that overexpression of HSPA8 inhibits the activation of the NLRP3 inflammasome.

In sepsis, the suppression of HSPA8 mediates the activation of NLRP3 inflammasome by attenuating ubiquitination modification level of NLRP3.

**Fig. 4:**
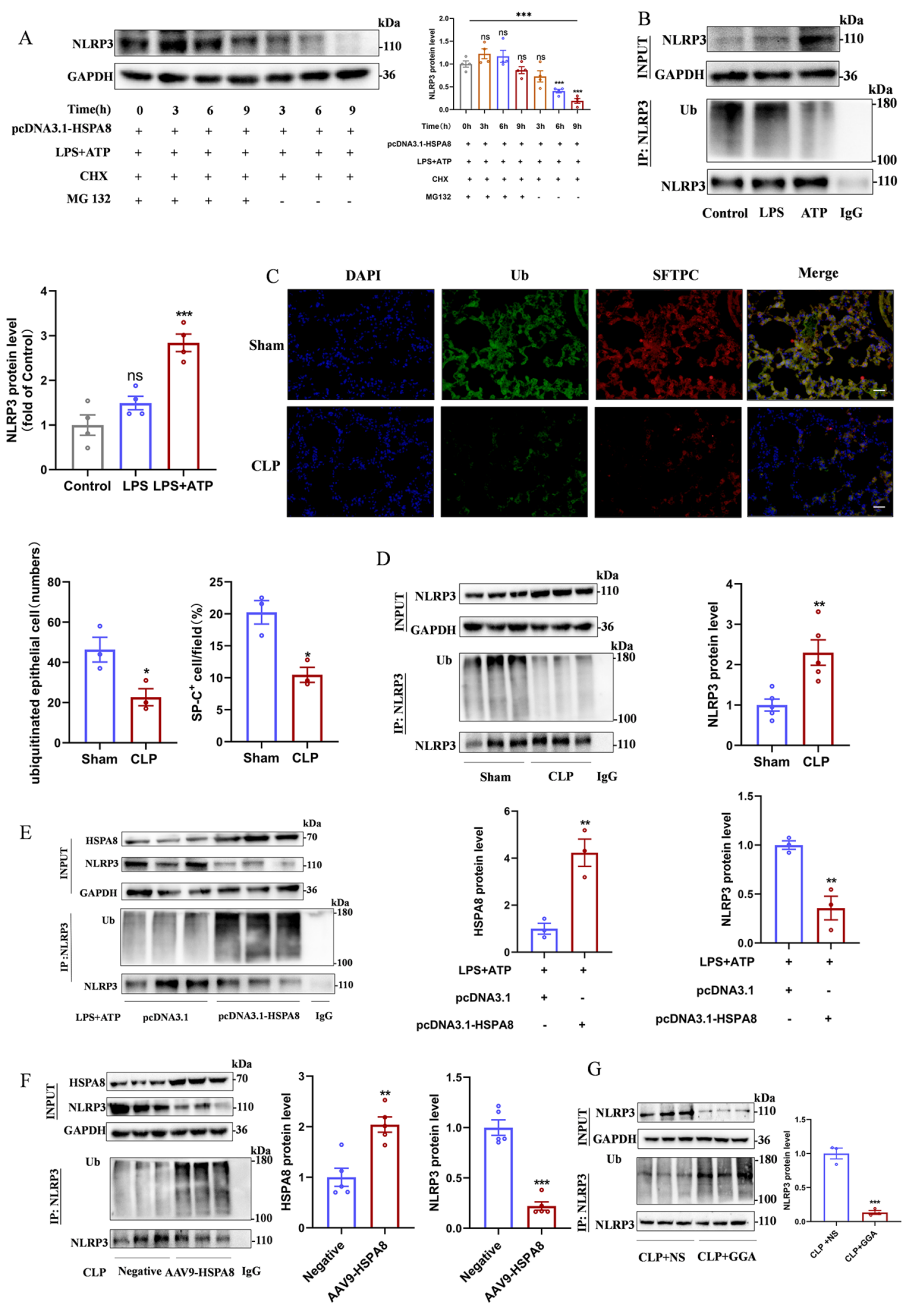
In sepsis, down-regulation of HSPA8 reduced NLRP3 ubiquitination levels. (**A**) Western blot analysis of NLRP3 protein expression after inhibition of ubiquitin proteasome by MG132 (*n* = 4). (**B**) Co-IP detection of NLRP3 ubiquitination level in control, LPS and LPS + ATP groups of MLE12 cells (*n* = 3). (**C**) Immunofluorescence representative images of lung tissue from Sham and CLP mice with double staining of epithelial-specific marker antibodies and ubiquitin antibodies (*n* = 3, scale = 50 μm). (**D**) The level of NLRP3 ubiquitination in lung tissue of Sham and CLP mice was detected by Co-IP (*n* = 3). (**E**) The level of NLRP3 ubiquitination was detected by Co-IP after pcDNA3.1-HSPA8 plasmid was transfected into MLE12 cells (*n* = 3). (**F**) The level of NLRP3 ubiquitination in CLP mice transfected with AAV9-HSPA8 was detected by Co-IP (*n* = 5). (**G**) The level of NLRP3 ubiquitination in lung tissue of GGA-pretreated CLP mice was detected by Co-IP (*n* = 3). Data are expressed as mean ± SEM, *p < 0.05, **p < 0.01, ***p < 0.001, ns means no significance

The results of CHX experiment showed that compared with the pcDNA3.1, the protein level of NLRP3 in the pcDNA3.1-HSPA8 decreased with the extension of CHX treatment (Fig. S3A). To determine the type of NLRP3 modification regulated by HSPA8, we transfected MLE12 cells with pcDNA3.1-HSPA8 and then treated them with the proteasome inhibitor MG132 for 12 h, followed by the intracellular protein synthesis inhibitor cycloheximide (CHX) for 3 h, 6 h, and 9 h, respectively. Western blot demonstrated that protein expression of NLRP3 compared with MG132 treatment group, MG132 untreated group in 3 h, 6 h, 9 h significantly decreased (Fig. 4A). Co-IP indicated that LPS + ATP increased the protein level of NLRP3 in MLE12 cells, while the ubiquitination level was decreased (Fig. 4B). In addition, the immunofluorescence results of lung tissue sections also suggested that the level of ACE II ubiquitination in the CLP group was significantly lower than that in the Sham group (Fig. 4C). This result was further confirmed by Co-IP experiments in lung tissues of sham and CLP mice (Fig. 4D). In sepsis, the increase of NLRP3 protein level is related to its ubiquitination modification.

To investigate the role of HSPA8 in the regulation of NLRP3 ubiquitination levels, we transfected MLE12 cells with pcDNA3.1-HSPA8 overexpression plasmid and treated CLP mice with AAV9-HSPA8 and GGA. The results showed that HSPA8 overexpression could increase the ubiquitination modification level of NLRP3 and reduce its protein level (Fig. 4E-G). Thus, we further confirmed that downregulation of HSPA8 in sepsis activated the NLRP3 inflammasome by reducing the ubiquitination level of NLRP3.

### HSPA8 promotes alveolar epithelial cell pyroptosis by interacting with SKP2 during sepsis

**Fig. 5:**
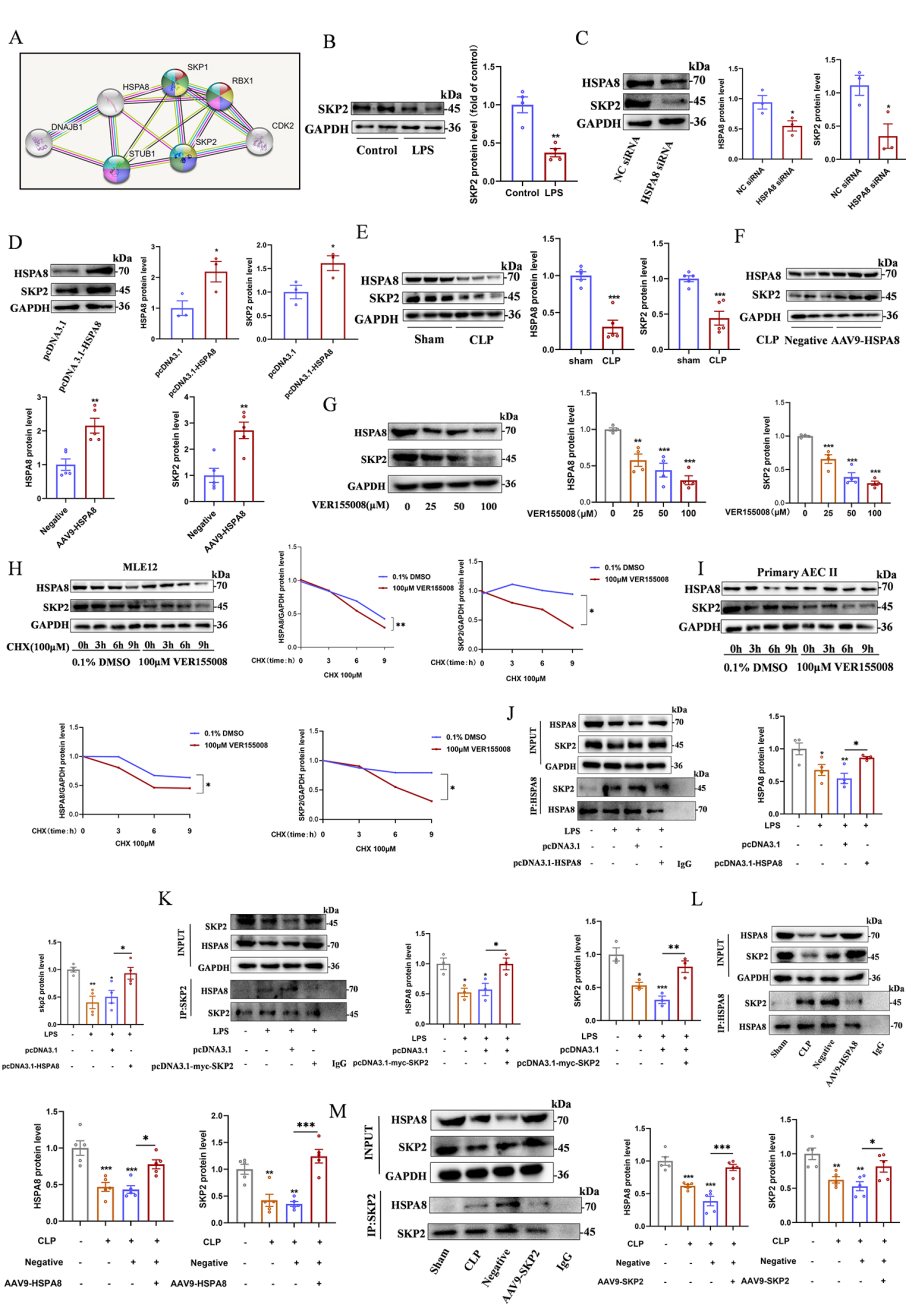
In sepsis, the suppression of HSPA8 promotes the degradation of the E3 ubiquitin ligase SKP2. (**A**) Diagram of the results of STRING protein interaction website analysis. (**B**) Western blot analysis of SKP2 protein expression in control and LPS in MLE12 cells (*n* = 4). (**C**) Western blot analysis of HSPA8 and SKP2 protein expression in MLE12 cells after transfection with HSPA8 siRNA (*n* = 4). (**D**) Western blot analysis of HSPA8 and SKP2 protein levels in MLE12 cells after transfection with pcDNA3.1-HSPA8 overexpression plasmid (*n* = 4). (**E**) Western blot analysis of HSPA8 and SKP2 protein levels in lung tissues of mice in Sham and CLP groups (*n* = 5). (**F**) Western blot analysis of HSPA8 and SKP2 protein levels in lung tissue of CLP mice infected with AAV9-HSPA8 (*n* = 5). (**G**) Western blot analysis of HSPA8 and SKP2 protein levels in MLE12 cells treated with different concentrations of HSPA8 inhibitor VER155008 (*n* = 4). (**H**) Western blot analysis the protein half-life levels of HSPA8 and SKP2 in MLE12 cells treated with HSPA8 inhibitor VER155008 and CHX at different times (*n* = 4). (**I**) Western blot analysis the protein half-life levels of HSPA8 and SKP2 in primary ACE II treated with HSPA8 inhibitor VER155008 and CHX at different times (*n* = 4). (**J**-**K**) The relationship between HSPA8 and SKP2 in control, LPS, LPS + pcDNA3.1 and LPS + PCDNA3.1-HSPA8 groups in MLE12 cells was detected by Co-IP (*n* = 3–4). (**L** - **M**) The relationship between HSPA8 and SKP2 in sham, CLP, negative control and AAV9-HSPA8 groups was detected by Co-IP (*n* = 5). Data are expressed as mean ± SEM, *p < 0.05, **p < 0.01, ***p < 0.001, ns means no significance

The results of STRING website analysis showed that HSPA8 had a regulatory effect on SKP2, which was mainly related to ubiquitination (Fig. 5A). In order to further explore the mechanism of HSPA8 in regulating the NLRP3 inflammasome, we designed experiments to examine the interaction between HSPA8 and its downstream E3 ubiquitin ligase SKP2. When MLE12 cells were stimulated with LPS, the protein level of SKP2 was significantly decreased (Fig. 5B). Knockdown of HSPA8 by siRNA reduced the level of SKP2, while overexpression of HSPA8 further increased the level of SKP2 (Fig. 5C and D). Compared with Sham group, HSPA8 and SKP2 in lung tissue of CLP group were simultaneously decreased, while SKP2 protein levels were also increased after AAV9-HSPA8 was transfected (Fig. 5E-F). Western blot demonstrated that a dose-dependent decrease in SKP2 protein levels when MLE12 cells were treated with different concentrations of VER155008 (Fig. 5G). In addition, when intracellular protein synthesis was stimulated by VER155008 and inhibited by CHX in all MLE12 and primary ACE II cells, the half-life of SKP2 protein was significantly reduced in the VER155008 group compared with DMSO treatment (Fig. 5H-I). Meanwhile, Co-IP showed that compared with the control, the interaction between HSPA8 and SKP2 was significantly enhanced in the LPS and significantly decreased in the pcDNA3.1-HSPA8 (Fig. 5J-K). AAV9-HSPA8/AAV9-SKP2 infected CLP mice to detect the interaction between HSPA8 and SKP2. The results demonstrated that the interaction between HSPA8 and SKP2 was enhanced in the CLP group, but decreased in the AAV9-HSPA8/AAV9-SKP2 group (Fig. 5L-M). In sepsis, HSPA8 interacts with the E3 ubiquitin ligase SKP2, and the down-regulation of HSPA8 promotes the degradation of SKP2.

In sepsis, suppression of the E3 ubiquitin ligase SKP2 activates the NLRP3 inflammasome by attenuating the level of ubiquitination modification of NLRP3.

**Fig. 6:**
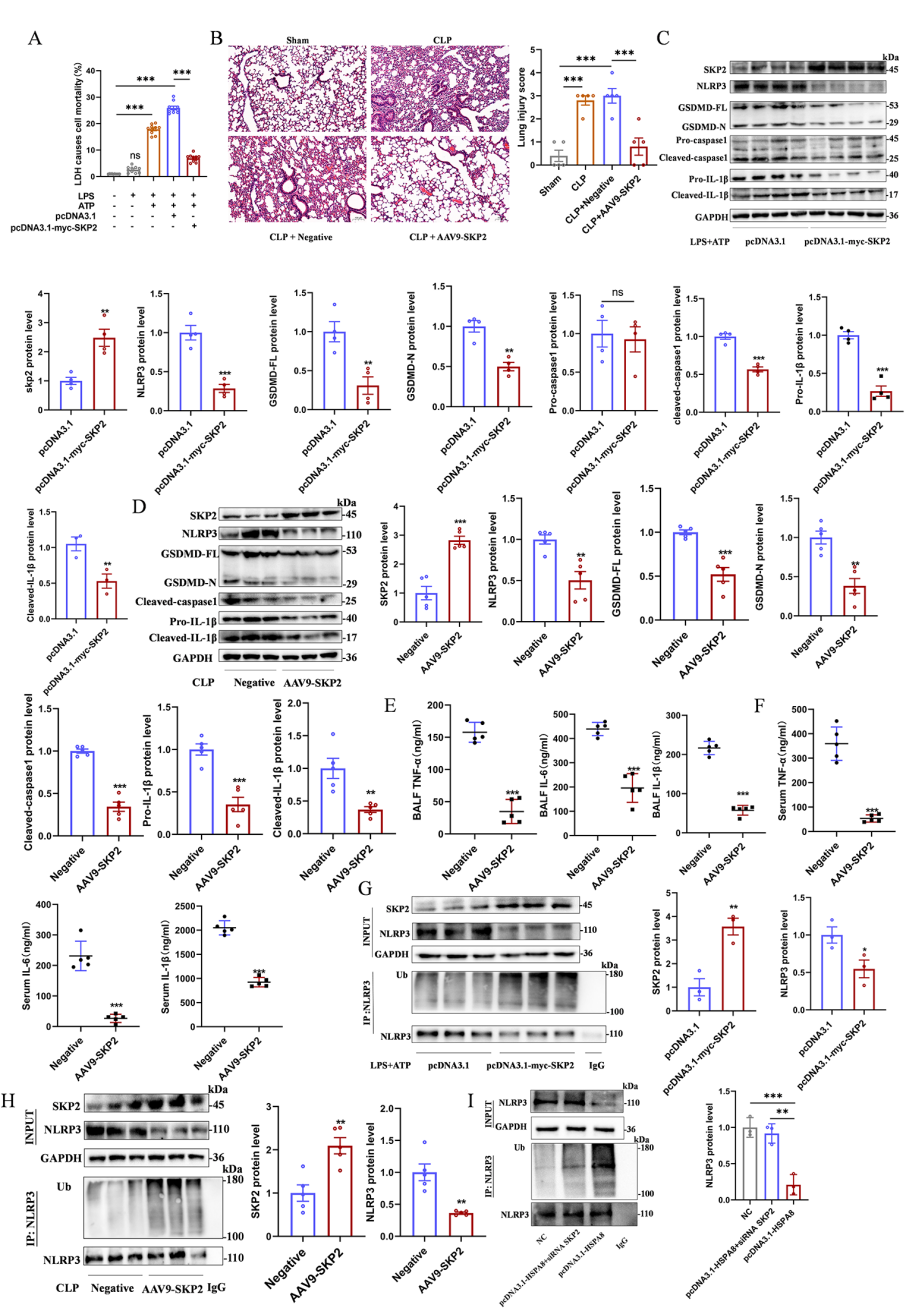
In sepsis, inhibition of SKP2 promotes inflammasome activation by reducing the ubiquitination level of NLRP3. (**A**) Comparison of LDH release levels in MLE12 cells treated with control, LPS, LPS + ATP, LPS + ATP + pcDNA3.1 and LPS + ATP + pcDNA3.1-myc-SKP2 (*n* = 10). (**B**) Representative H&E staining and histological scores of lung section in Sham, CLP, negative control and AAV9-SKP2 (*n* = 5, scale = 100 μm). (**C**) Western blot analysis of the expression of inflammasome activation-related proteins in MLE12 cells transfected with pcDNA3.1-myc-SKP2 plasmid (*n* = 4). (**D**) Western blot analysis of the expression of inflammasome activation-related proteins in CLP infected by AAV9-SKP2 (*n* = 5). (**E**) ELISA analysis of BALF in Sham, CLP, Negative and AAV9-SKP2 groups (*n* = 5). (**F**) ELISA analysis of serum in Sham, CLP, Negative and AAV9-SKP2 groups (*n* = 5). (**G**) The level of NLRP3 ubiquitination in MLE12 cells transfected with pcDNA3.1-myc-SKP2 plasmid was detected by Co-IP(*n* = 3). (**H**) The level of NLRP3 ubiquitination in CLP mice infected by AAV9-SKP2 was detected by Co-IP (*n* = 5). (**I**). Western blot analysis of NLRP3 protein and ubiquitination levels after co-transfection of pcDNA3.1-HSPA8 and siRNA SKP2 in MLE12 cells(*n* = 3). Data are expressed as mean ± SEM, *p < 0.05, **p < 0.01, ***p < 0.001, ns means no significance

To evaluate the effect of E3 ubiquitin ligase SKP2 on NLRP3 inflammasome mediated pyroptosis, LDH release levels were detected. The results showed that the LPS + ATP group had a significantly higher level of LDH release than the control and LPS, while the pcDNA3.1-myc- SKP2 group had a lower LDH release level (Fig. 6A). H&E staining suggested that the degree of lung injury in CLP mice treated with AAV9-SKP2 was significantly reduced (Fig. 6B). Western blot was used to analyze the activation of NLRP3 inflammasome in MLE12 cells transfected with pcDNA3.1-myc-SKP2. The results showed that compared with pcDNA3.1 group, the protein levels of NLRP3, GSDMD-N, GSDMD-FL, Cleaved-caspase1, Pro-IL-1β and Cleaved-IL-1β in pcDNA3.1-myc-Skp2 group were significantly decreased. However, there was no significant difference in Pro-caspase1 protein levels (Fig. 6C). Similarly, AAV9-SKP2 was used to detect the activation of NLRP3 inflammasome in the lung tissue of CLP mice, and the same experimental results were obtained (Fig. 6D). ELISA also showed that the released levels of TNF-α, IL-6, and IL-1β from AAV9-SKP2 BALF and serum were significantly reduced compared with the negative control (Fig. 6E-F). Further study showed that the ubiquitination modification level of NLRP3 in pcDNA3.1-myc-SKP2 was significantly higher than that in control group (Fig. 6G). The same results were obtained when AAV9-SKP2 was infected in CLP mice (Fig. 6H). In addition, MLE12 cells were co-transfected with pcDNA3.1-HSPA8 and siRNA SKP2 to detect the protein and ubiquitination levels of NLRP3. The results showed that compared with the NC group, the protein level and ubiquitination level of NLRP3 in pcDNA3.1-HSPA8 + siRNA SKP2 group did not change significantly, while the NLRP3 protein level of pcDNA3.1-HSPA8 was significantly decreased, and the ubiquitination level was significantly increased (Fig. 6I). These results suggest that attenuation of the E3 ligase SKP2 activated the NLRP3 inflammasome by inhibiting the level of ubiquitination modification of NLRP3.

### In sepsis, the E3 ubiquitin ligase SKP2 has a regulatory effect on NLRP3

**Fig. 7:**
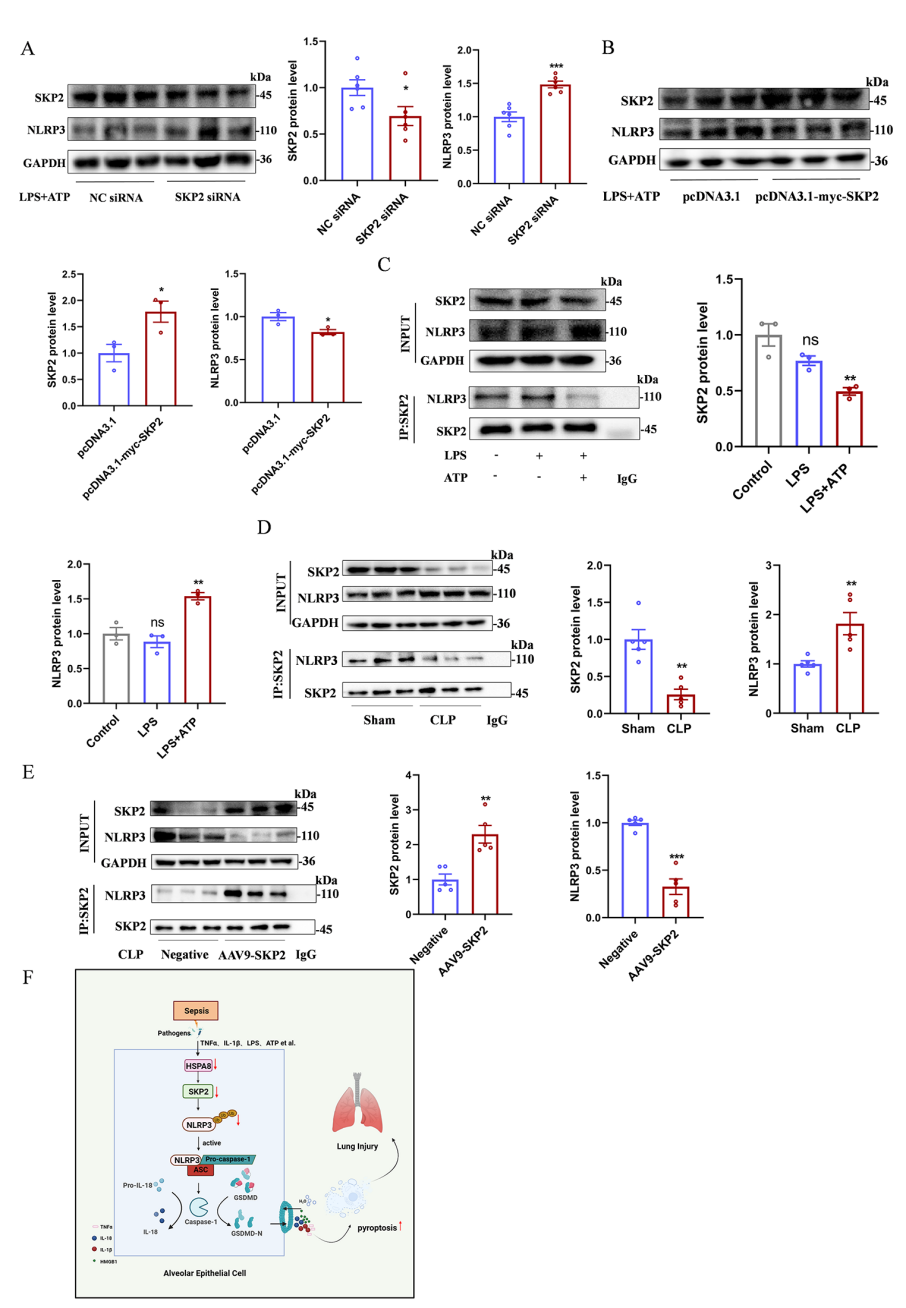
In sepsis, the interaction between the E3 ubiquitin ligase SKP2 and NLRP3 is reduced. (**A**) Western blot analysis of SKP2 and NLRP3 protein levels in MLE12 cells after transfection with SKP2 siRNA (*n* = 6). (**B**) Western blot analysis of SKP2 and NLRP3 protein levels in MLE12 cells after transfection with pcDNA3.1-myc-SKP2 overexpression plasmid (*n* = 3). (**C**) The relationship between SKP2 and NLRP3 in MLE12 control group, LPS group and LPS + ATP group was detected by Co-IP (*n* = 4). (**D**) The relationship between SKP2 and NLRP3 in lung tissue of sham and CLP groups was detected by Co-IP(*n* = 5). (**E**) The relationship between SKP2 and NLRP3 in lung tissue of negative control and AAV9-SKP2 transfected CLP mice was detected by Co-IP(*n* = 5). (**F**) Schematic diagram of HSPA8 mediating pyroptosis in AECs by promoting SKP2 degradation and inhibiting NLRP3 ubiquitination during sepsis. Data are expressed as mean ± SEM, *p < 0.05, **p < 0.01, ***p < 0.001, ns means no significance

To verify the interaction between SKP2 and NLRP3, MLE12 cells were first transfected with SKP2 siRNA, which showed that the NLRP3 protein level was increased compared with the control group (Fig. 7A). NLRP3 protein levels were significantly decreased when SKP2 was overexpressed (Fig. 7B). Co-IP showed that the interaction between SKP2 and NLRP3 in LPS + ATP group was significantly decreased, and the protein level of NLRP3 was significantly increased compared with control and LPS group (Fig. 7C). Similar results were obtained in animal experiments (Fig. 7D). In addition, Co-IP assay further showed that the effects of SKP2 and NLRP3 in the CLP group infected with AAV9-SKP2 were significantly enhanced compared with the negative control, while the NLRP3 protein level was significantly decreased (Fig. 7E). These data suggest that SKP2 has a regulatory role on NLRP3 in sepsis.

## Discussion

ALI occurs earliest in the process of sepsis and is the leading cause of hospitalization and death in sepsis [18]. Irreversible damage to AECs leads to barrier dysfunction and uncontrolled inflammatory responses that ultimately cause lung injury [20, 21]. Previous studies have shown that caspase11-mediated pyroptosis of endothelial cells can cause lung injury in LPS-challenged mice [21]. In sepsis, mitochondrial aldehyde dehydrogenase 2 (ALDH2) promotes the activation of NLRP3 inflammasome and participates in the occurrence of pyroptosis [22]. Present study found that LPS + ATP stimulation can promote the activation of NLRP3 inflammasome in AECs and mediate the occurrence of pyroptosis. We show that NLRP3 inflammasome activation could be caused by HSPA8-induced degradation of the E3 ubiquitin ligase SKP2, which results in reduced NLRP3 ubiquitination. Meanwhile, overexpression of HSPA8 can significantly inhibit the pyroptosis of AECs stimulated by LPS + ATP, thereby alleviating lung injury during sepsis (Fig. 7F). These findings provide a new perspective for further understanding the development of sepsis and potential therapeutic targets for the management of sepsis-induced lung injury.

In the heat shock protein superfamily, HSP70 is involved in the occurrence of sepsis-induced lung injury [23]. HSPA8, a member of HSP70 family, is constitutively expressed in cells. Although HSPA8 and HSP70 are similar in structure and function, they are fundamentally different [24]. This study is the first to focus on the role of HSPA8 in regulating NLRP3 inflammasome activation in AECs in the development of sepsis-induced lung injury. Traditionally, HSPA8 is considered to be a stress-protective protein. When the body is under heat stress, viral infection, and trauma, the level of HSPA8 rapidly increases to maintain the homeostasis of the body, while the level of HSPA8 decreases due to persistent inflammation and trauma. This may be related to HSPA8 translocation, oxidative stress or post-translational modification [8, 25, 26]. Consistent with these results, we found that HSPA8 protein levels increased rapidly at 3 h of LPS stimulation in AECs, then began to decrease at 6 h and continued to decrease at 12 h and 24 h. Previous studies have shown that HSP70 can play a protective role in ALI in sepsis by interacting with KANK2 to reduce the release of apoptosis-inducing factor and apoptosis [27]. Further studies suggested that HSPA12A deficiency aggravated LPS-induced primary hepatocyte injury [28]. In this study, we provided evidence that the suppression of HSPA8 promoted death in CLP mice when HSPA8 protein levels were inhibited by VER155008, while GGA-induced HSPA8 expression improved survival in septic mice. We also found that, compared with the control, pyroptosis indicators in AECs, as well as lung injury were significantly reduced in the CLP mice infected with AAV9-HSPA8. Taken together, these results suggest that HSPA8 could prevent septic lung injury.

SKP2 is a subunit of the SCF ubiquitin E3 ligase complex. SKP2 has the activity of an E3 ubiquitin ligase and acts as a substrate recognition factor to promote ubiquitination of target proteins. Previous studies have demonstrated that SKP2 promotes Akt K63-mediated ubiquitination, enhancing the interaction between Akt and HK2, leading to increased cisplatin resistance in NPC [29]. Further studies have found that HSP70 inhibition promotes the degradation of SKP2, which affects the cell cycle and promotes apoptosis of gastric cancer cells [30]. Moreover, The STRING protein interaction website analysis results suggested that main role of HSPA8 was to mediate the ubiquitination and degradation of proteins, and HSPA8 had a protein-protein interaction with E3 ubiquitin ligase SKP2-SCF complex, and this interaction was mainly related to the positive regulation of protein ubiquitination and degradation. However, the regulatory mechanism of HSPA8 on SKP2 in sepsis-induced lung injury remains unclear. In present study, we showed that when HSPA8 protein levels in sepsis were knockdown by siRNA or inhibited by VER155008, SKP2 protein levels were subsequently reduced. Vice versa, overexpression of HSPA8 with pcDNA3.1-HSPA8 plasmid or AAV9-HSPA8 increased the protein level of SKP2. Co-IP assay showed that there was a significant interaction between HSPA8 and SKP2 in sepsis. The results of CHX assay also showed that inhibition of HSPA8 promoted the degradation of SKP2 and significantly shortened its half-life. This suggests that HSPA8 downregulation plays a role by promoting the degradation of SKP2 in sepsis. Furthermore, overexpression of SKP2 with pcDNA3.1-myc-SKP2 or AAV9-SKP2 not only inhibited NLRP3 inflammasome activation but also reduced GSDMD-N levels. Co-IP experiment showed that there was an interaction between SKP2 and NLRP3. This suggests that HSPA8 knockdown, by promoting the degradation of SKP2, causes its protein levels to decrease. As an E3 ubiquitin ligase, the decreased protein level of SKP2 further inhibits the ubiquitination level of NLRP3, which increases its protein level and activates NLRP3 inflammasoma-mediated pyroptosis in septic alveolar epithelial cells. We verified that HSPA8 inhibited sepsis-induced lung injury by promoting NLRP3 ubiquitination by constructing pcDNA3.1-HSPA8 and adeno-associated virus AAV9-HSPA8. To make the downstream effects caused by overexpression of HSPA8 more convincing, we added experiments in which CLP mice and cells were treated with the agonist GGA and the inhibitor VER155008 of HSPA8. Meanwhile, in order to increase the reliability of the results, we also designed qRT-PCR, immunofluorescence, immunohistochemistry and other experimental methods for comprehensive verification.

Risk signals such as infection, tissue damage and metabolic disorders can activate the innate immune signaling receptor NLRP3 in cells. Once this signal is activated, NLRP3 aggregates into the inflammasome, leading to caspase1-mediated proteolytic activation of IL-1β family enzymatic cytokines, mediating activation of the IL-1β cytokine family, and inducing inflammatory pyroptotic cell death [31]. Abnormal activation of NLRP3 inflammasome is associated with a variety of diseases, such as non-alcoholic fatty liver disease [32], breast cancer [33], and atherosclerosis [34]. Although current studies have demonstrated the potential of NLRP3 as a drug target, there is no complete understanding of the structure and activation mechanism of NLRP3, hindering he development of new drugs against this target. As the research furthering, an emerging view is that the activation of NLRP3 inflammasome is precisely regulated by post-translational modification, especially ubiquitination modification [35]. NLRP3 is deubiquitinated upon initiation and activation, which is a key step in the formation and activation of the NLRP3 inflammasome [36]. According to previous studies, UAF1 deubiquitinase complex promotes NLRP3 inflammasome activation by inhibiting NLRP3 ubiquitination [37]. Although some E3 ubiquitin ligases have been reported, such as March7 [38], Ubc13 [39], TRIM31 [40] inhibit the activation of NLRP3 inflammasome by mediating the degradation of NLRP3 protein. Whether SKP2, as an E3 ubiquitin ligase, has a regulatory role in NLRP3 ubiquitination is currently unknown. In this study, we show that SKP2, as an E3 ubiquitin ligase, its suppression attenuate NLRP3 protein ubiquitination and subsequently promote NLRP3 inflammasome activation in sepsis. Overexpression of SKP2 at the cellular level or AAV9-SKP2 at the animal level promoted the ubiquitination and degradation of NLRP3 and inhibited the occurrence of pyroptosis. It can significantly alleviate the severity of lung injury. This suggests that the regulation of NLRP3 ubiquitination by SKP2 is involved in the process of lung injury in sepsis.

In conclusion, the present study demonstrates that the suppression of HSPA8 promotes the degradation of SKP2, which in turn attenuates the ubiquitination and degradation of NLRP3 protein, leading to the activation of NLRP3 inflammasome and subsequent AECs pyroptosis in during sepsis. These findings provide strong evidence for HSPA8 as a potential therapeutic target and suggest novel therapeutic strategies for managing sepsis-induced lung injury.

### Electronic supplementary material

Below is the link to the electronic supplementary material.

Supplementary Material 1

## Data Availability

Data will be made available on request.
