# Supplementary material for: The suppression of HSPA8 attenuates NLRP3 ubiquitination through SKP2 to promote pyroptosis in sepsis-induced lung injury
Source: Cell Biosci. 2024 May 2;14:56. doi: 10.1186/s13578-024-01239-z (PMC11064404; doi:10.1186/s13578-024-01239-z)
Supplement: Supplementary file 1 — Supplementary Material 1 [file 13578_2024_1239_MOESM1_ESM.docx]

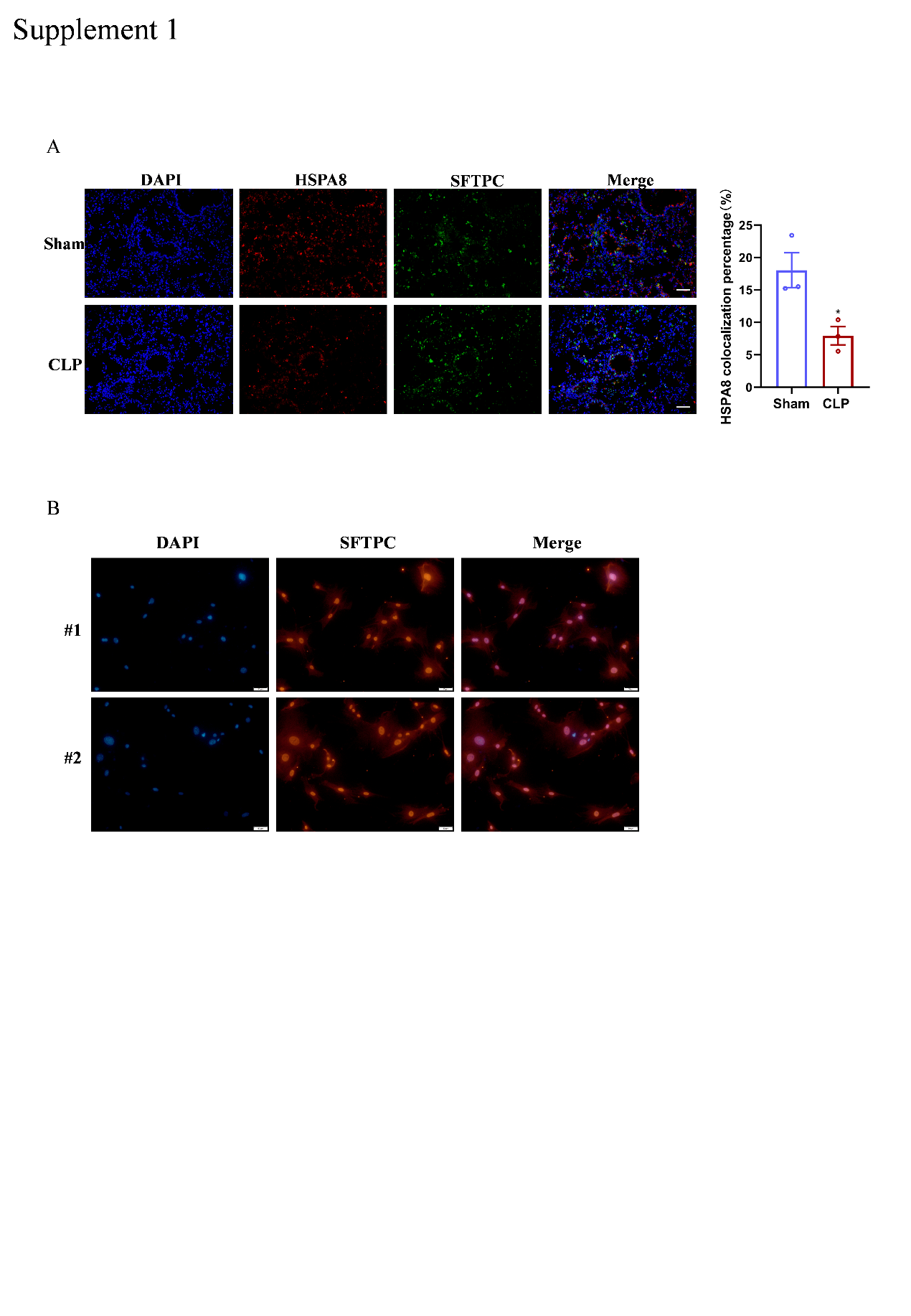


Fig. S1. (A) Representative images of immunofluorescence for HSPA8 and SFTPC double-staining in Sham and CLP (n=3, scale =100μm). (B) Immunofluorescence representative images of primary AEC II extracted from the lungs of C57 mice (n=4, scale =50μm)


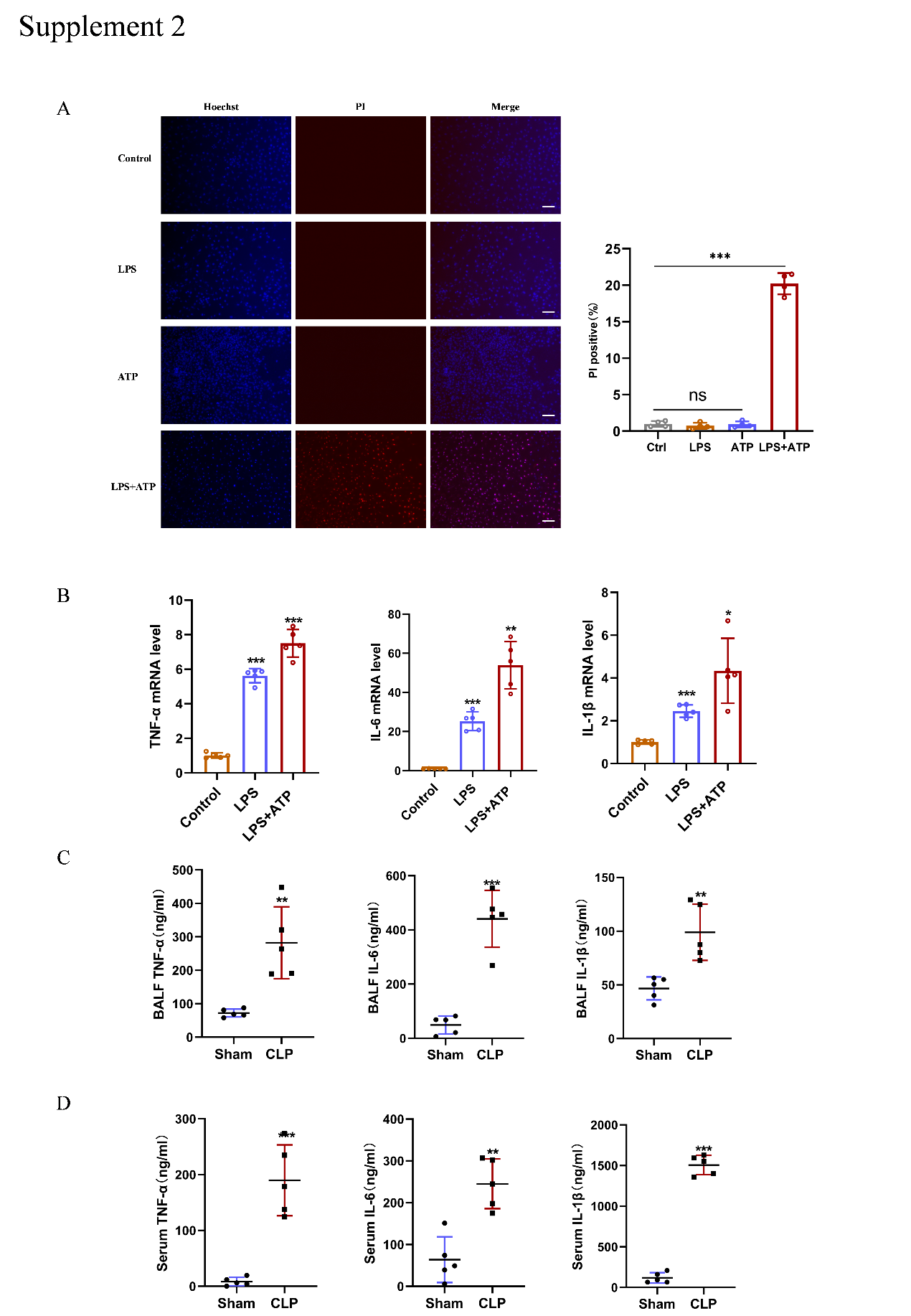


Fig. S2. (A) Hoechst33342/PI staining of MLE12 cells stimulated by ctrl, LPS, ATP, and LPS+ATP (n=4, scale =50μm). (B) mRNA levels of TNF-α, IL-6 and IL-1β in MLE12 cells induced by Control, LPS and LPS+ATP (n=5). (C) ELISA analysis of BALF TNF-α, IL-6 and IL-1β levels in Sham and CLP mice (n=5). (D)ELISA analysis of Serum TNF-α, IL-6 and IL-1β levels in Sham and CLP mice (n=5).


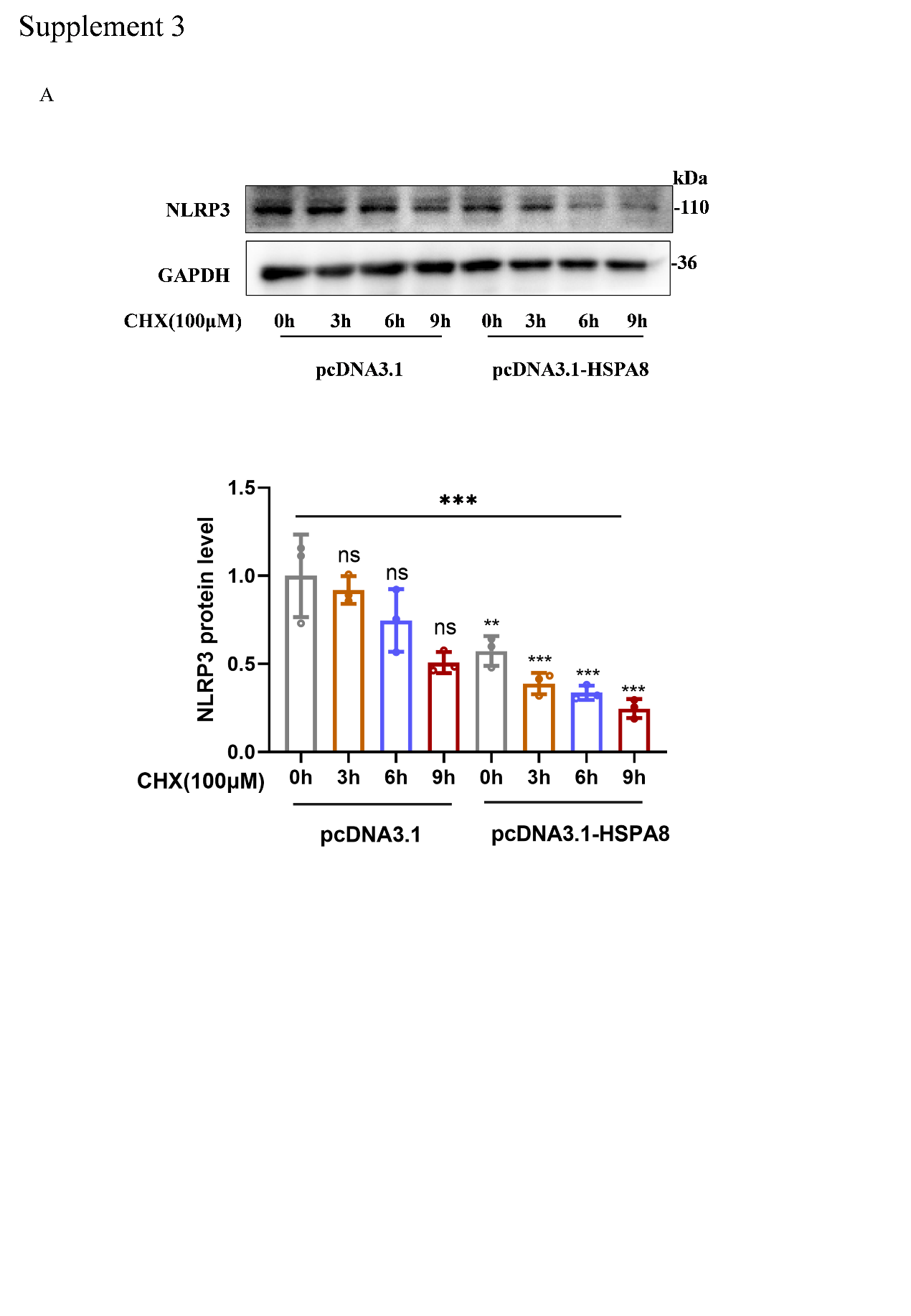


Fig. S3. （A）Western blot detection pcDNA3.1 - HSPA8 plasmid transfection MLE12 cells after NLRP3 protein levels(n=3).
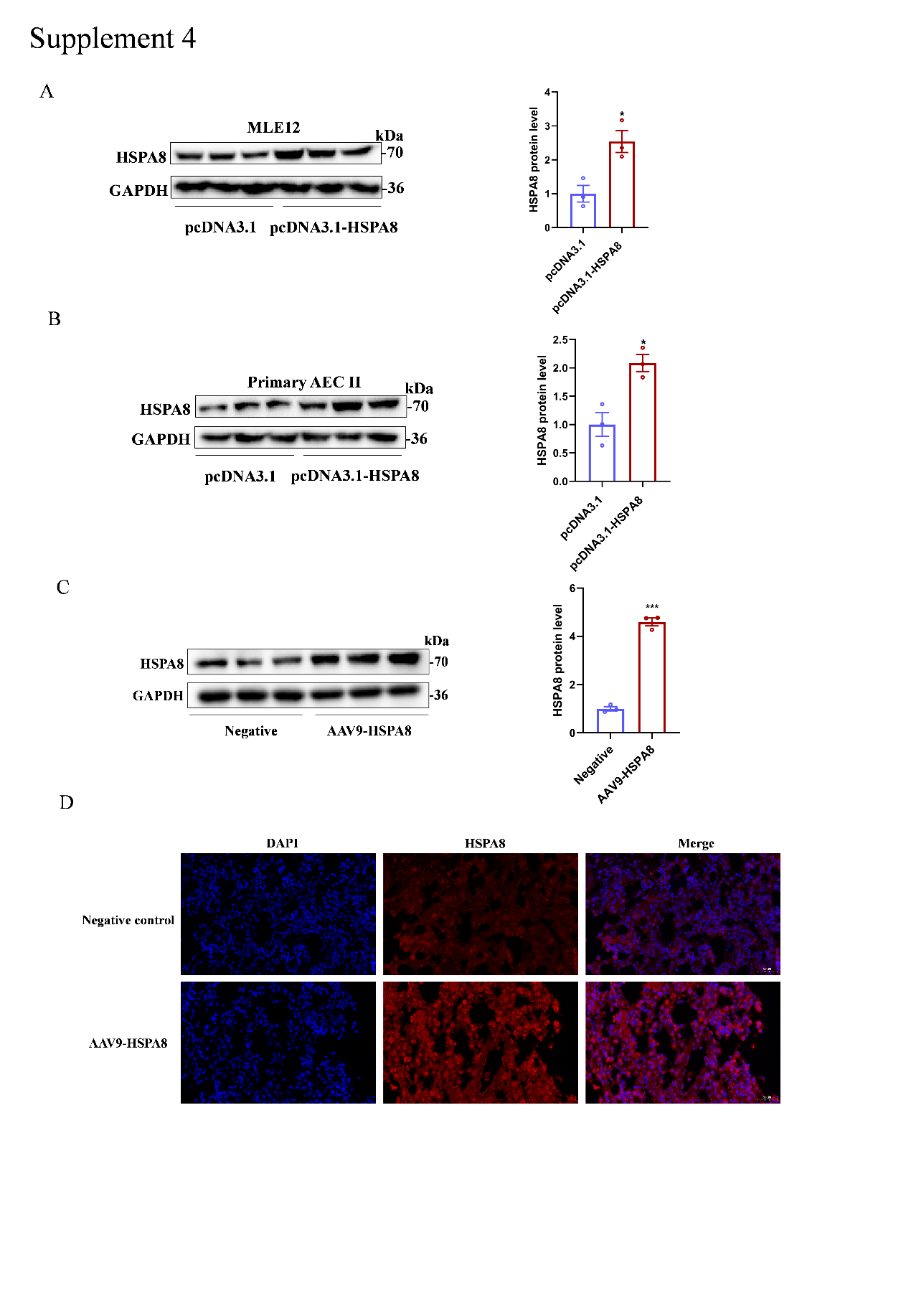


Fig. S4. (A) Western blotting analysis of the overexpression efficiency of pcDNA3.1-HSPA8 plasmid transfection in MLE12 cells (n=3). (B) Western blotting analysis of the overexpression efficiency of pcDNA3.1-HSPA8 plasmid transfection in primary AECs (n=3). (C) Western blotting analysis of the overexpression efficiency of AAV9-HSPA8 infection in CLP mice (n=3). (D) Immunofluorescence representative of lung tissue of AAV9-HSPA8-infected CLP mice (n=3, scale=50μm).


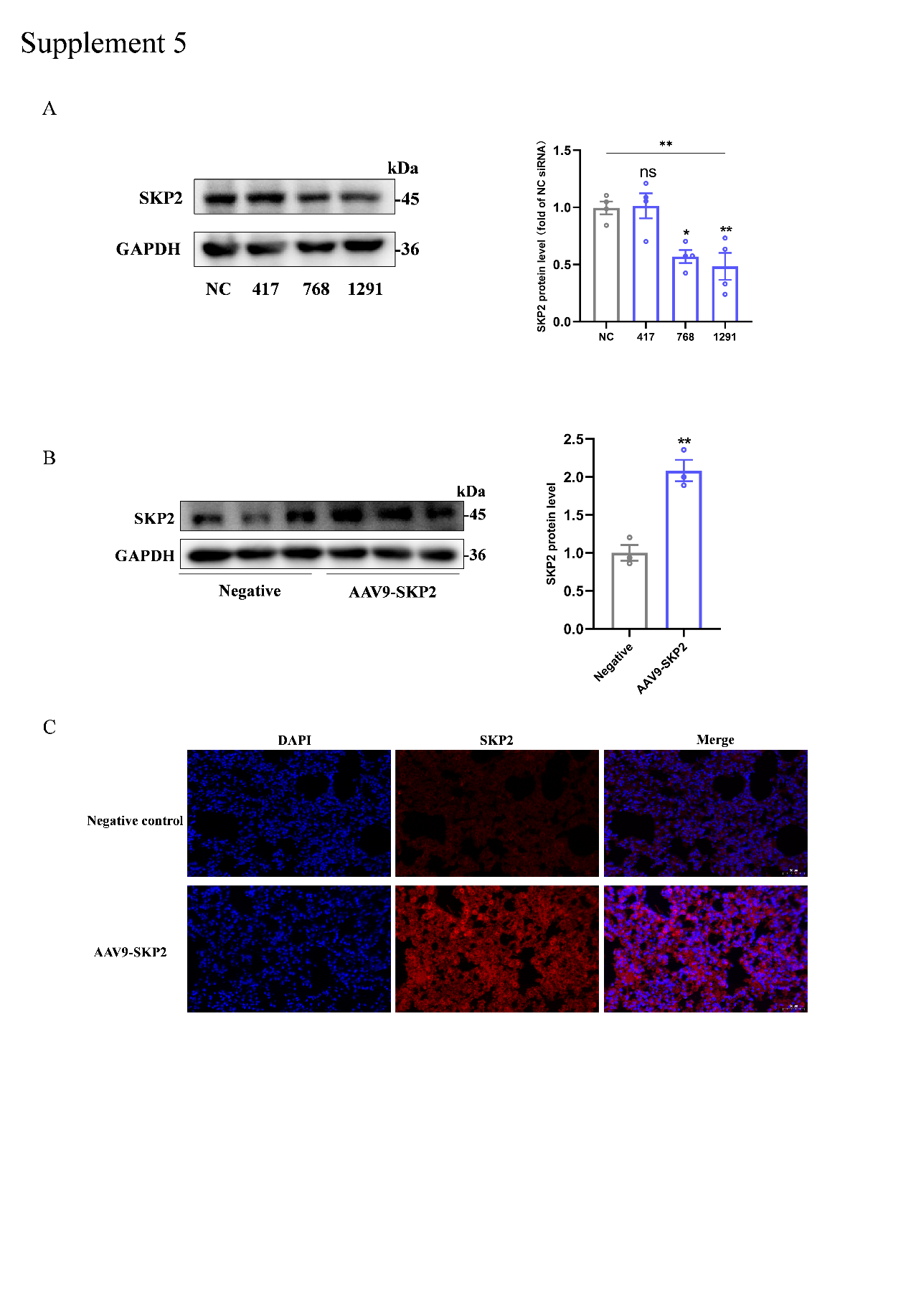


Fig. S5. (A) western blot analysis of knockdown efficiency of SKP2 siRNA transfected MLE12 cells (n=3). (B) Western blotting analysis of the overexpression efficiency of AAV9-SKP2 infection in CLP mice (n=3). (C) Immunofluorescence representative of lung tissue of AAV9-SKP2-infected CLP mice (n=3, scale=50μm).


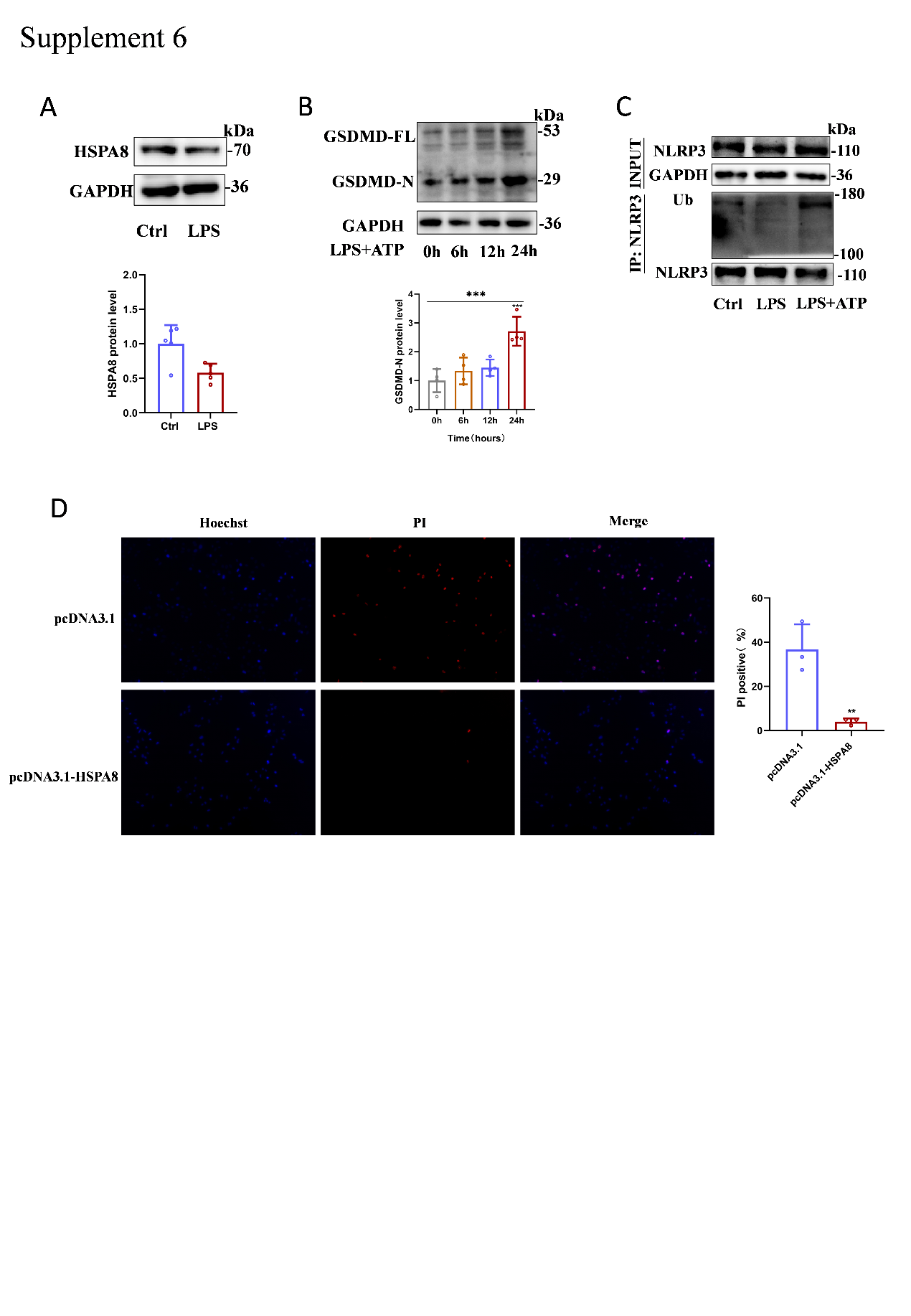


Fig. S6. (A)Western blot analysis of HSPA8 protein levels in LPS-stimulated A549 cells(n=4). (B) Western blot analysis of GSDMD-N protein levels in A549 cells stimulated by LPS+ATP at different times(n=4). (C) Representative plot of A549 cells after transfection of pcDNA3.1-HSPA8 by Hoechst33342/PI staining(n=3).


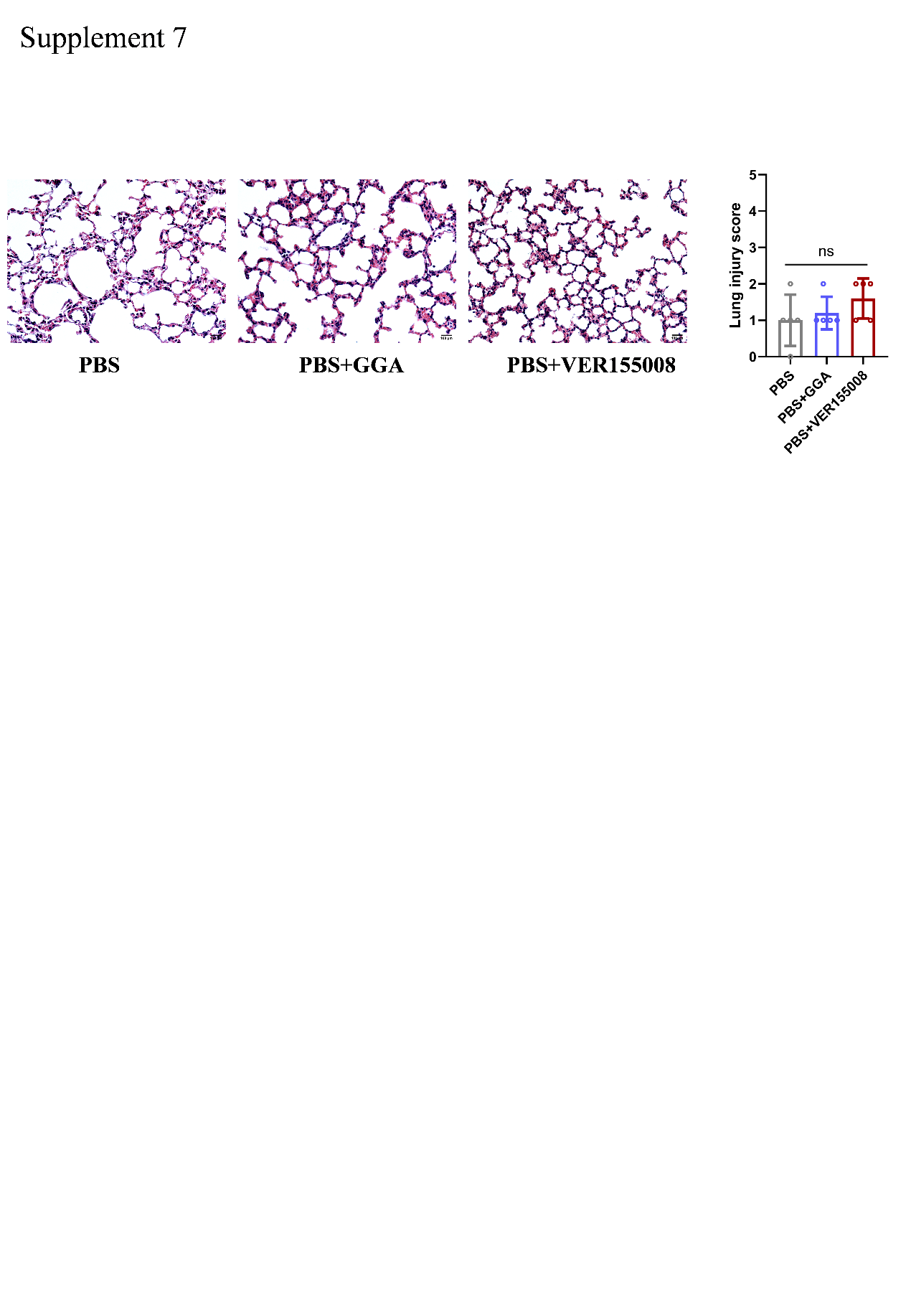


Fig.S7. Representative HE staining and lung injury score of normal C57 mice treated with GGA and VER155008 (n=5).
